# Effect of Free Nitrous Acid on Nitrous Oxide Production and Denitrifying Phosphorus Removal by Polyphosphorus-Accumulating Organisms in Wastewater Treatment

**DOI:** 10.1155/2018/9192607

**Published:** 2018-04-26

**Authors:** Zhijia Miao, Duo Li, Shan Guo, Zhirui Zhao, Xiaofeng Fang, Xueyou Wen, Jingmin Wan, Aiguo Li

**Affiliations:** ^1^School of Water Resources and Environment, Hebei GEO University, Shijiazhuang 050031, China; ^2^Hebei Province Key Laboratory of Sustained Utilization and Development of Water Resources, Shijiazhuang 050031, China; ^3^Hebei Province Collaborative Innovation Center for Sustainable Utilization of Water Resources and Optimization of Industrial Structure, Shijiazhuang 050031, China; ^4^Research Center of Natural Resources Assets, Hebei GEO University, Shijiazhuang 050031, China

## Abstract

The inhibition of free nitrous acid (FNA) on denitrifying phosphorus removal has been widely reported for enhanced biological phosphorus removal; however, few studies focus on the nitrous oxide (N_2_O) production involved in this process. In this study, the effects of FNA on N_2_O production and anoxic phosphorus metabolism were investigated using phosphorus-accumulating organisms (PAOs) culture highly enriched (91 ± 4%) in* Candidatus Accumulibacter phosphatis*. Results show that the FNA concentration notably inhibited anoxic phosphorus metabolism and phosphorus uptake. Poly-*β*-hydroxyalkanoate (PHA) degradation was completely inhibited when the FNA concentration was approximately 0.0923 mgHNO_2_-N/L. Higher initial FNA concentrations (0.00035 to 0.0103 mgHNO_2_-N/L) led to more PHA consumption/TN (0.444 to 0.916 mmol-C/(mmol-N·gVSS)). Moreover, it was found that FNA, rather than nitrite and pH, was likely the true inhibitor of N_2_O production. The highest proportion of N_2_O to TN was 78.42% at 0.0031 mgHNO_2_-N/L (equivalent to 42.44 mgNO_2_-N/L at pH 7.5), due to the simultaneous effects of FNA on the subsequent conversion of NO_2_ into N_2_O and then into N_2_. The traditional nitrite knee point can only indicate the exhaustion of nitrite, instead of the complete removal of TN.

## 1. Introduction

Enhanced biological phosphorus removal (EBPR), operated with sequential anaerobic and aerobic periods, is considered as an efficient method for phosphorus removal. A group of bacteria known as polyphosphate-accumulating organisms (PAOs) are able to take up volatile fatty acids (VFAs) and store them as poly-*β*-hydroxyalkanoates (PHAs), which has been attributed to phosphorus release during the anaerobic phase. In the subsequent aerobic phase, PAOs use the stored PHA as an energy source for biomass growth and take up orthophosphate into polyphosphate (poly-p). Finally, phosphorus is removed from the system through the wastage of excess sludge. Phosphorus uptake also occurs under anoxic conditions. Previous studies have identified a subset of PAOs, known as denitrifying phosphorus-accumulating organisms (DPAOs), which can also denitrify and are able to oxidize intracellular PHA for energy and nitrite or nitrate as electronic acceptors, instead of oxygen, to remove phosphorus [1–3]. Compared to conventional biological nitrogen and phosphorus removal, DPAOs can take up a carbon source during the anaerobic phase, which could be used for both denitrification and phosphorus removal. Thus, the DPAO pathway is advantageous for the treatment of wastewater containing a relatively low level of organic carbon, while requiring less oxygen and resulting in lower sludge production.

N_2_O, which is a significant greenhouse gas, has 300 times greater warming potential than CO_2_ (IPCC, 2001). Most research on the pathway of nitrification, denitrification, and phosphorus removal has been conducted that N_2_O was produced in wastewater treatment systems. The production of N_2_O may be affected by many parameters, such as low dissolved oxygen concentrations, accumulation of nitrite, types of organic carbon sources, pH, and temperature [4, 5]. It has been frequently reported that the accumulation of nitrite leads to increased N_2_O emission, rather than N_2_, as the major end-product in the denitrifying phosphorus removal processes [6, 7]. Zhou et al. showed that free nitrous acid (FNA), rather than nitrite or pH, is likely the true inhibitor of N_2_O reduction by DPAOs [8]. Wang et al. also observed the inhibitory effect of FNA on the N_2_O reduction activity in a denitrifying phosphorus removal system [9]. These results indicate that FNA is associated with denitrifying phosphorus removal and N_2_O production.

The level of the FNA can also affect the anoxic or aerobic phosphorus metabolism of PAOs. Zhou et al. reported that the concentration of FNA influenced the efficiency of anoxic phosphorus uptake. Specifically, the PAO uptake process was inhibited at lower FNA levels (<0.002 mg HNO_2_-N/L) and ceased at an FNA concentration of 0.02 mg HNO_2_-N/L [10]. It was found that FNA inhibited all key aerobic metabolic processes performed by a culture highly enriched (90 ± 5%) in* Candidatus Accumulibacter phosphatis* [11]. In contrast, some studies revealed that PAOs could acclimate to nitrite as the sole electron acceptor without experiencing inhibition [12, 13].

Most studies in this field have taken great efforts to understand the key roles of FNA in the metabolic processes of PAOs. Previous research has shown that N_2_O production accompanies denitrifying phosphorus removal; however, little attention has been given to the effect of nitrite/FNA on N_2_O production by highly enriched PAOs. The PAOs, named* Candidatus Accumulibacter phosphatis*, are dominant in both lab-scale EBPR reactors and full-scale wastewater treatment plants (WWTPs) [14–17]. If PAOs contribute to N_2_O emission, we cannot ignore their role in WWTP operations. Therefore, the impact of nitrite/FNA on N_2_O production in anoxic denitrifying phosphorus removal by PAOs should be further investigated.

In this study, a series of batch tests were carried out using a highly enriched culture of* Candidatus Accumulibacter phosphatis* under different pH values and nitrite concentrations. The objective was to determine the effects of FNA on PHA oxidation, nitrite reduction, phosphorus uptake, and N_2_O accumulation during denitrifying phosphorus removal and to determine whether these effects are associated with N_2_O metabolism.

## 2. Methods

### 2.1. Reactor and Operation

A laboratory-scale sequence batch reactor (SBR) with a working volume of 8 L was operated for 241 days under anaerobic-aerobic conditions. The SBR was fed with acetate or propionate switching at a frequency of one to two sludge ages. The cycle time was 6 h and consisted of a 150 min anaerobic period, a 180 min aerobic period, 25 min settle/decant period, and a 5 min idle period. In each cycle, 2 L of synthetic wastewater was fed to the reactor in the first 6 min of the anaerobic period, resulting in a hydraulic retention time (HRT) of 24 h. At the end of the cycle, 200 ml sludge was removed to achieve a solids retention time (SRT) of approximately 10 days and a mixed liquor suspended solid (MLSS) level of 2.5–3.5 g/L. The dissolved oxygen (DO) concentration was maintained at 2.0 ± 0.2 mg/L in aerobic period, using an online on/off controller. The pH was controlled during both the anaerobic and aerobic phases at the range of 7.2–8.0 through dosed 0.5 MHCL and 0.5 MNaOH. The temperature was maintained at 20°C.

### 2.2. Synthetic Wastewater

The 2 L synthetic wastewater described by Miao et al. was composed of 0.3 L of solution A and 1.7 L of solution B [18]. The mixed feed of solutions A and B contained 800 COD/L and 40 mgP·L^−1^. Solution A contains (per liter) 3.41 g acetate or 1.76 ml propionic acid. In addition, solution A also contains (per liter) 1.02 g NH_4_Cl, 0.01 g peptone, 0.01 g yeast extraction, 1.20 gMgSO_4_·7H_2_O, 0.19 g CaCl_2_·2H_2_O, 7.94 mgallyl-N thiourea (ATU, a nitrification inhibitor), and 4.00 mL trace elements liquid. Solution B contained (per liter) 173 mg K_2_HPO_4_·3H_2_O and 104 mg KH_2_PO_4_. For the propionic acid feed, 10.47 ml of 5 M NaOH was used to adjust the pH to 7.5.

### 2.3. Batch Experiments

#### 2.3.1. Batch Experiment 1

The tested sludge was taken from a SBR that was fed with acetate during the normal cycle. At the end of the anaerobic stage, the mixed liquor (6 L) was divided into six parts and put into a 1.25 L batch reactor. NaNO_2_ (0.1971 g) was added to the batch reactors under different pH conditions, resulting in the initial nitrite concentrations varying between 39.98 and 48.63 mg NO_2_-N/L. FNA concentration, pH, temperature, and anoxic operating time are shown in Table 1. The concentration of FNA (HNO_2_-N) was calculated according to the following formula:

(1) $$\begin{array}{c} FNA=\frac{S_{\left({{NO}_{2}}^{-}\text{–}N\right)}}{K_{a}\times 10^{pH}},\;K_{a}=e^{-2300/\left(273+T\right)} \end{array}$$

(see [19]). In each set, the pH was kept approximately constant (±0.1) at a set-point, by manually adding 0.2 M HCl or 0.2 M NaOH.

#### 2.3.2. Batch Experiment 2

The tested sludge was taken from a SBR at the end of the anaerobic stage. Six sets of batch tests were performed similar to that described above. The details are shown in Table 1.

### 2.4. Analytical Methods

The liquid samples were immediately filtered through Millipore filter units (0.45 *μ*m pore size) for analysis of COD, VFA, NO_2_-N, NO_3_-N, and PO_4_-P. During the experiment, COD, NO_2_-N, and PO_4_-P were measured according to standard methods (APHA, 1998).

PHA analysis was performed using the method of Miao et al. to determine poly-*β*-hydroxybutyrate (PHB), poly-*β*-hydroxyvalerate (PHV), and poly-*β*-hydroxy-2-methylvalerate (PH2MV) [18]. Weighed freeze-dried biomass, 2 ml chloroform, and 2 ml methanol acidified with 3% H_2_SO_4_ were added to glass tubes, respectively, and then the tubes were heated in 100°C for 20 h after being mixed. One millilitre of Milli-Q water was put into the tubes and mixed after cooling. After centrifugation, 1400 ml of the bottom organic phases was added to GC vial for analysis. The temperatures of injector and FID detector were maintained at 200°C and 250°C. The temperature program was set as follows: it was held at 80°C for 2 min; increased to 140°C at the rate of 10°C/min, and then maintained for 1 min. Glycogen was measured according to the method described by Lopez-Vazquez. Five millilitres of 0.6 M HCl was added to weighed freeze-dried biomass in crew-toped glass tubes and then heated at 105°C for 6 h [20]. After cooling and centrifugation, 1 ml of the supernatant was transferred to a high performance liquid chromatography vial for glucose analysis. N_2_O analysed was using the N_2_O sensor by microsensor multimeter.

Fluorescence in situ hybridization (FISH) was performed with cy5-labelled EUBMIX probes (for most bacteria), cy3-labelled GAOMIX probes (for competibacter, comprising equal amounts of probes GAO431 and GAO 989), and cy3-labelled PAOMIX (for* Candidatus Accumulibacter phosphatis *or* Accumulibacter*, comprising equal amounts of probes PAO462, PAO651, and PAO846) [21, 22].

## 3. Results and Discussion

### 3.1. Reactor Performance and Microbial Community

**Figure 1:**
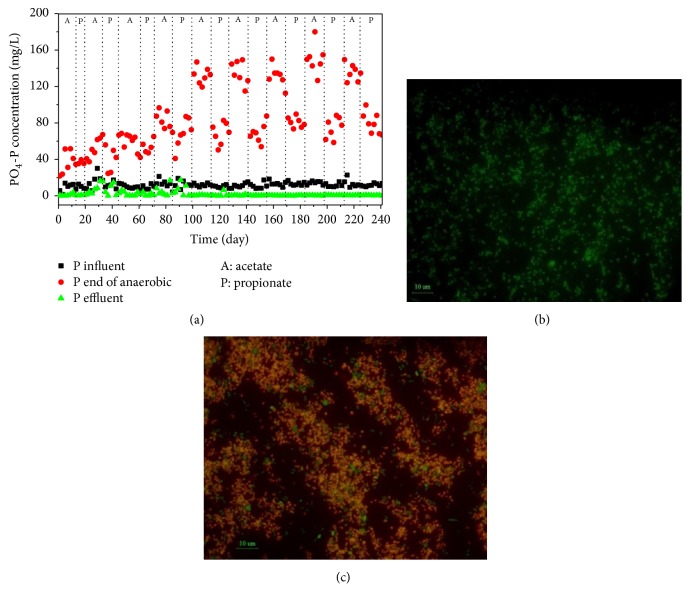
Performance of the reactor and FISH results: (a) Phosphorus removal performance; (b) FISH image of day 1; (c) FISH image of day 241.

The SBR was operated for 241 days under anaerobic/aerobic conditions. The reactor was fed with acetate or propionate, which was switched at a frequency of one to two sludge ages. The phosphorus removal performance is shown in Figure 1(a). During the first 45 days, the phosphorus removal efficiency was not stable, with the P concentration higher in the effluent than in the influent for some days. The pump supplying the carbon source malfunctioned from days 83 to 85, leading to a shortage of carbon sources during the anaerobic metabolic process; thus, the P removal efficiency declined in the effluent. After the 95th day, the P concentration in the effluent was stably maintained at less than 0.8 mg/L for the remainder of the operation. The P concentration at the end of the anaerobic stage averaged 130 mg/L when fed with acetate, which was higher than when fed with propionate as the carbon source (approximately 80 mg/L). The composition of the microbial population was characterised using FISH (Figures 1(b) and 1(c)). FISH results showed that the abundance of* Accumulibacter* was initially 3% of the total bacteria on day 1 and then rose to 91% (±4%) on day 241. The glycogen accumulating organisms (GAOs) were hardly detected on the 241st day. After 241 days of sludge domestication, the fraction of PAOs could utilize nitrite instead of oxygen as electron acceptor was to be found. In addition, high N_2_O accumulation occurred during the denitrifying phosphorus removal process. One possible explanation was that FNA affected this phase. Thus, batch experiments 1 and 2 were designed to investigate the influence of FNA on anoxic phosphorus metabolism by PAOs.

### 3.2. Effect of FNA on Denitrifying Phosphorus Removal and N_2_O Production in Batch Experiment 1

#### 3.2.1. Comparison of Nitrite Reduction in Batch Experiment 1

**Figure 2:**
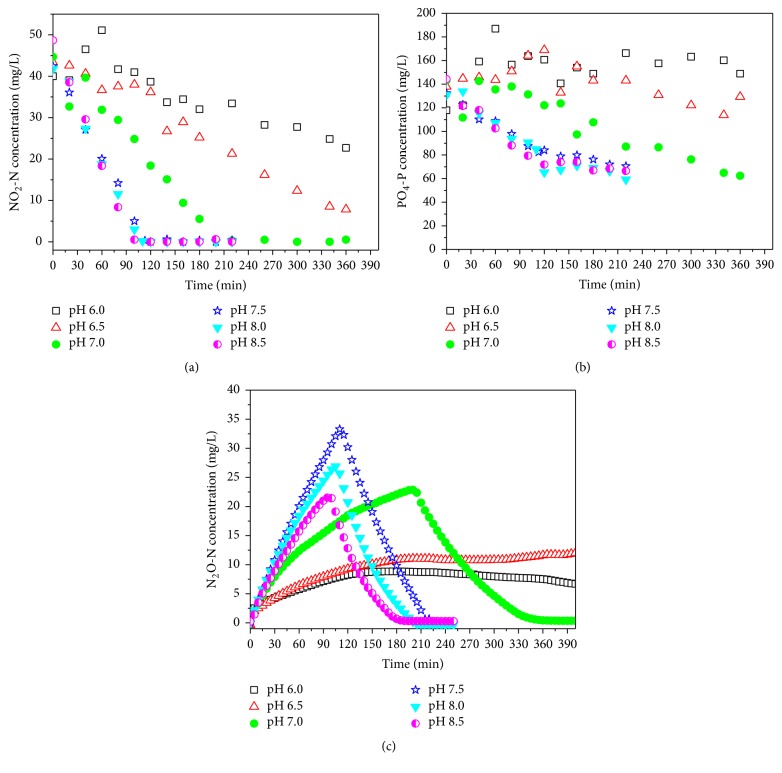
Profiles of nitrite, phosphorus, and nitrous oxide: (a) nitrite reduction; (b) phosphorus uptake; (c) N_2_O production. (pH of 6.0, 6.5, 7.0, 7.5, 8.0, and 8.5 corresponds to 0.0923, 0.0316, 0.0103, 0.0031, 0.00096, and 0.00035 mgHNO_2_-N/L, resp.).

The variations in nitrite concentrations during the anaerobic denitrifying phosphorus process were monitored throughout batch experiment 1 (Figure 2(a)). Following addition of 40 mgN/L nitrite, the nitrite concentration decreased rapidly at the ranges of 0.0031, 0.00096, and 0.00035 mgHNO_2_-N/L (corresponding to pH 7.5, 8.0, and 8.5 conditions, resp.) (Figure 2(a)), thereby resulting in nitrite reduction rates of 8.15, 8.25, and 11.7 mgNO_2_-N/(h·gVSS), respectively. With the decrease of FNA, the nitrite reduction rate slowed and reached 1.16, 2.14, and 4.82 mgNO_2_-N/(h·gVSS), corresponding to 0.0923, 0.0316, and 0.0103 mgHNO_2_-N/L. In several studies the denitrifying phosphorus removal process was restrained at lower nitrite concentrations from approximately 5 to 10 mgN/L [3, 23]. In this study, nitrite addition of approximately 40 mg NO_2_-N/L was not inhibitory to the anoxic metabolism of the PAOs at the FNA concentrations of 0.0103, 0.0031, 0.00096, and 0.00035 mgHNO_2_-N·L^−1^. However, the nitrite reduction rate was inhibited at FNA concentrations of 0.0923 and 0.0316 mgHNO_2_-N/L. This result suggests that nitrite is not a main inhibitor of the denitrifying phosphorus removal process when 39.98–48.63 mg NO_2_-N/L was added at the beginning of the experiment. Our findings are consistent with the research conducted by Zhou et al., who reported that FNA, rather than nitrite, was likely the true inhibitor of anoxic phosphorus uptake [10]. In that study, the concentration ranged from 0.002 to 0.02 mg HNO_2_-N/L and anoxic phosphorus uptake ceased at 0.02 mg HNO_2_-N/L. In this study, nitrite reduction occurred at an FNA concentration of 0.0923 mgHNO_2_-N·L^−1^. A possible explanation is that the higher proportion of PAOs (91% (±4%) of the total bacteria) would be tolerant of higher FNA concentrations.

#### 3.2.2. Comparison of Phosphorus Uptake in Batch Experiment 1

Figure 2(b) shows the phosphorus concentration profiles measured in batch experiment 1. Similar to the nitrite reduction rate, phosphorus uptake occurred rapidly at the ranges of 0.0031, 0.00096, and 0.00035 mgHNO_2_-N/L, and the average rates were determined as 6.13, 8.10, and 8.63 mgP/(h·gVSS), respectively. The phosphorus concentration decreased rapidly during the initial stage under these three conditions, with the turning point occurring when the terminal nitrite concentration was less than 0.5 mgN/L. However, the rate decreased sharply after the turning point (2.418, 3.686, and 3.715 mgP/(h·gVSS)), when N_2_O replaced nitrite as the sole electronic acceptor. The concentration of phosphorus slightly decreased when FNA declined to 0.0316 mgHNO_2_-N/L. Moreover, denitrifying phosphorus removal was completely inhibited at 0.0923 mgHNO_2_-N/L, suggesting that phosphorus release occurs instead of phosphorus uptake under this condition. This observation confirms that PAO metabolism during the anoxic phosphorus removal process may be inhibited by FNA and that poly-P would be released from intracellular or dead biomass under these circumstances.

**Figure 3:**
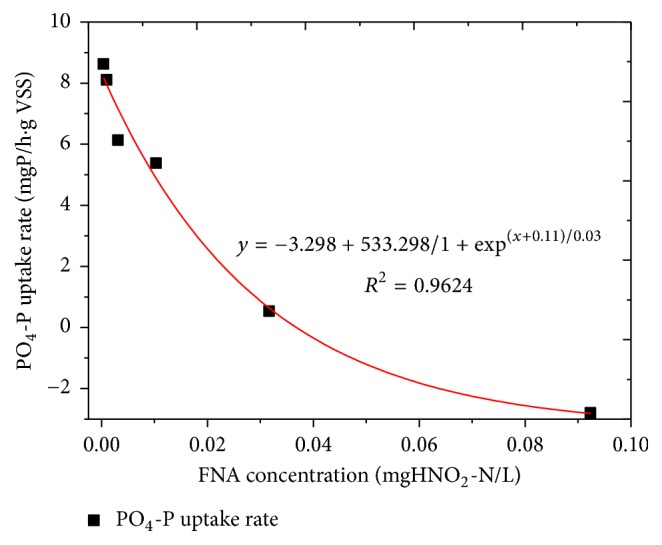
The inhibitory effect of FNA on PAO activity and P uptake rates.

Figure 3 shows that the average P uptake rate significantly correlated with the FNA concentration, indicating the importance of FNA in the denitrifying phosphorus process. The rates were between −2.08 and 8.63 mgP/(h·gVSS), which is higher than some previously published research [10]. However, these rates were still lower than using oxygen as the electron acceptor when operated at normal cycle (data not shown). Therefore, it could be deduced that the nitrite is denitrified by PAOs when the concentration of FNA is below inhibitory levels.

#### 3.2.3. Comparison of N_2_O Production in Batch Experiment 1

High concentrations of N_2_O accumulated during the denitrifying phosphorus removal process by PAOs, with the fact that the concentration of N_2_O peaked when nitrite was exhausted (Figure 2(c)). This result suggests that it has different reduction rates between the different steps during the anoxic phosphorus removal process, namely, NO_2_^−^ to N_2_O and N_2_O to N_2_, thereby leading to N_2_O accumulation. Some studies have shown that nitrite reductase and N_2_O reductase play important roles in these two steps of denitrifying metabolism [24, 25]. Recently, Zhou et al. demonstrated that the N_2_O reduction rate seemed to be independent of pH, whereas the inhibitory effect was much more moderate in comparison to that of FNA, when using denitrifying enhanced biological phosphorus removal sludge [8].

**Figure 4:**
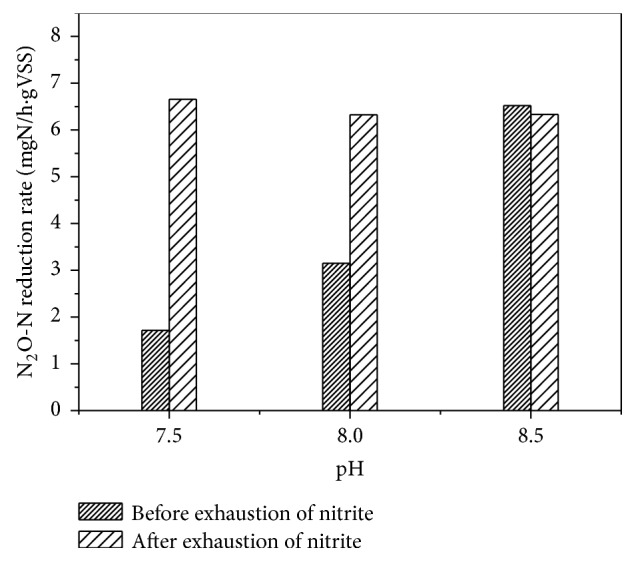
Various N_2_O reduction rates in the presence or absence of nitrite (pH of 7.5, 8.0, and 8.5 corresponds to 0.0031, 0.00096, and 0.00035 mgHNO_2_-N/L).

In batch experiment 1, at a similar nitrite concentration in the initial anoxic phase, the N_2_O reduction rate was different before and after the exhaustion of nitrite (Figure 4). After nitrite was exhausted, the N_2_O reduction rates were similar (6.65, 6.33, and 6.33 mgN/(h·gVSS)) despite different pH conditions when the N_2_O was not detected in the reactor at 107 min, 90 min, and 80 min, respectively. However, before the exhaustion of nitrite, the rise in FNA concentrations resulted in the decrease of N_2_O reduction rates (1.72, 3.15, and 6.52 mgN/(L·g VSS)), at the corresponding times of 113 min, 110 min, and 100 min. This may indicate that the N_2_O reduction rate was mainly affected by FNA in the denitrifying phosphorus removal process. In contrast, as shown in Table 2 and Figure 2(a), FNA also inhibited the NO_2_^−^ to N_2_O step (nitrite reduction rate), which resulted in the highest N_2_O accumulation occurring at 0.0031 mg HNO_2_-N/L (corresponding to pH 7.5). The proportion of highest N_2_O accumulation to TN was from 18.8 to 78.42%.

#### 3.2.4. Comparison of PHA Consumption Performance in Batch Experiment 1

The type of carbon source is important in influencing N_2_O production under anoxic conditions. In the current study, PHA played a key role in N_2_O production during denitrifying phosphorus removal [9]. In this study, the carbon source for denitrification was PHB and PHV. The surplus levels of PHB and PHV at the end of the anoxic phase were above 58%. This suggests that a shortage of carbon source was not a main inhibitory factor for the metabolic process. The PHA degradation rates for concentrations of 0.0316, 0.0103, 0.0031, 0.00096, and 0.00035 mgHNO_2_-N/L were 0.067, 0.278, 0.589, 0.581, and 0.603 mmol-C/(h·g VSS), respectively, before exhaustion of nitrite in the reactor (Table 2). However, PHA was synthesized instead of being degraded at 0.0923 mgHNO_2_-N/L. The PHA degradation rate sharply decreased from 0.0106 mgHNO_2_-N/L to 0.0316 mgHNO_2_-N/L. Some studies suggest that FNA has a stronger inhibitory effect on energy-consuming, rather than energy-generating, processes by DPAOs [26, 27]. In this study, the process related to energy generation, namely, PHA degradation, was seriously inhibited at high FNA levels and completely collapsed at 0.0923 mgHNO_2_-N/L.

The quantity of PHA consumption per nitrite in the denitrifying phosphorus removal process by PAOs is shown in Table 2. Only the data that operated within the range of 0.00035 to 0.0103 mgHNO_2_-N/L are listed, due to the complete consumption of TN during the processes above. The levels of PHA/TN per biomass were 0.444, 0.532, 0.546, and 0.916 mmol-C/(mmol-N·gVSS), respectively. Therefore, we could infer that a higher FNA concentration would lead to more PHA consumption during the initial anoxic phase. A possible explanation is that FNA could passively diffuse across the cell membrane and shuttle protons between the two sides without generating energy, which may induce the collapse of the proton motive force (PMF); therefore, the cell should pump out more protons in order to resist this trend [8, 28]. In other words, more PHA was consumed to provide energy and protons to maintain the PMF balance or to recover from the inhibition that resulted from the absence of nitrite, when anoxic phosphorus removal occurred at the higher initial FNA concentration.

### 3.3. Batch Experiment 2

In batch experiment 1, the increase in FNA concentrations resulted in a decrease of the nitrite reduction rate, phosphorous uptake and N_2_O reduction. N_2_O accumulated due to the inhibition of the N_2_O reduction rate. However, further evidence was needed to demonstrate whether FNA was more crucial than pH. In batch experiment 2, various quantities of nitrite were added to the PAO sludge at two pH levels to test effects of FNA on N_2_O reduction rates and phosphorus uptake rates (Table 1).

**Figure 5:**
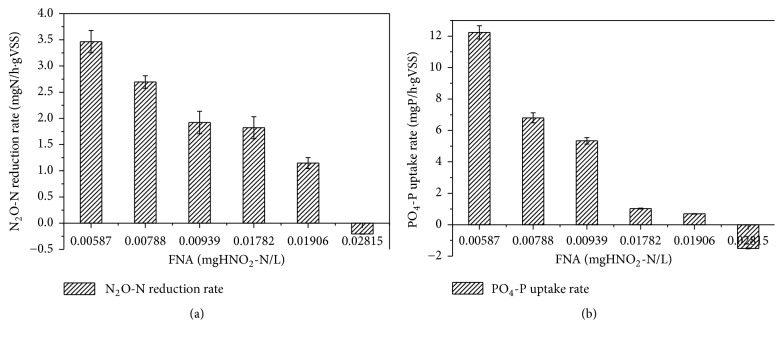
Effects of FNA on N_2_O reduction rates and phosphorus uptake rates in batch experiment 2: (a) N_2_O reduction rate; (b) phosphorus uptake rate.

At pH 7.5, the N_2_O reduction rates were 3.465, 2.695, and 1.92 mgN/(h·gVSS), while the PO_4_-P uptake rates were 12.24, 6.80, and 5.34 mgP/(h·gVSS) (Figures 5(a) and 5(b)). Both rates decreased under the same pH conditions. The average value of the maximum N_2_O reduction rates and PO_4_-P uptake rates decreased by 44.6% and 56.4%, respectively, at which time the FNA concentration increased from 0.01781 to 0.02814 mgHNO_2_-N/L. Meanwhile, similar trends were observed for pH 7.0. The results provide evidence that higher initial FNA concentrations, not pH, cause a lower N_2_O reduction rate and phosphorus uptake rate in denitrifying phosphorus removal by PAOs. Therefore, FNA has an important effect on anoxic phosphorus removal and on N_2_O production. This finding is consistent with results from Wang et al., who reported that nitrite addition to DPAO sludge stimulated the net N_2_O production rate due to FNA inhibition [9]. Zhou et al. reported a 50% inhibition at FNA concentrations between 0.0007 and 0.001 mgHNO_2_-N/L, while the procedure was carried out to an FNA concentration of 0.004 mgHNO_2_-N/L [8]. In our study, FNA concentrations ranged from 0.00587 to 0.02815 mgHNO_2_-N/L (Table 1), and the total inhibitory concentration was 0.02815 mgHNO_2_-N/L, providing strong evidence that FNA has a main inhibitory effect on the N_2_O reduction rate, resulting in accumulation of N_2_O.

### 3.4. The Performance of ORP in Batch Experiment 1

**Figure 6:**
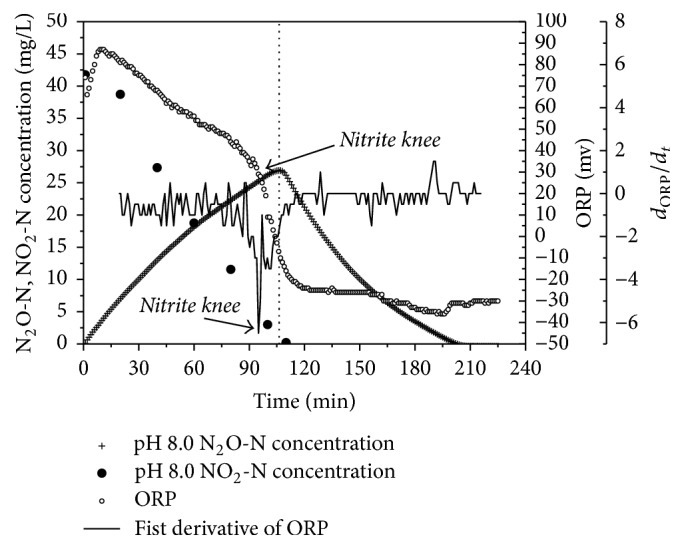
Various ORP in denitrifying phosphorus removal.

Many studies have reported that Oxidation-Reduction Potential (ORP) is the main parameter for determining the nitrate or nitrite depletion point during anoxic nitrogen removal [29, 30]. The nitrate or nitrite knees were a flex with a negative slope in the ORP curve and were more clearly shown in the sharply decreasing value of *d*_ORP_/*d*_*t*_. The ORP curve at pH 8.0 during denitrifying phosphorus removal is shown in Figure 6; similar results were observed at pH 7.0, 7.5, and 8.5. The nitrite knee appeared at 99 min while the concentration of nitrite was 3 mg/L (Figure 6). It is feasible to consider that this value was a nitrite knee point according to the traditional explanation; however, TN still existed in the form of N_2_O at a concentration of 26.05 mg/L. Hence, using this nitrite knee to indicate the end of the denitrifying phosphorus removal process is not appropriate, and part of dissolved N_2_O could be diffused into the air if the denitrifying phosphorus removal immediately stopped at this time. Meanwhile, it is also difficult to use an N_2_O probe to indicate the end of denitrifying phosphorus removal due to its fragility. Further studies should focus on other efficiency parameters that can be used in denitrifying phosphorus removal.

## 4. Conclusions

The FNA concentration significantly influenced the denitrifying phosphorus removal process by PAOs when nitrite was added as the electron acceptor. The nitrite reduction rate, phosphorus uptake rate, N_2_O reduction rate, and PHA degradation rate also decreased as the concentration of FNA increased. FNA prevented the step from NO_2_ and N_2_O to N_2_O and N_2_. In addition, when the FNA concentration was approximately 0.0031 mgHNO_2_-N/L (equivalent to 42.44 mgNO_2_-N/L at pH 7.5), the accumulation of N_2_O approaches 33.28 mgN_2_O-N/L during the anoxic denitrification process, accounting for 78.42% of the total nitrogen. The traditional nitrite knee point can only indicate the exhaustion of nitrite, instead of the complete removal of TN.

## Figures and Tables

**Table 1:** Batch experiment conditions applied in batch tests.

	Batch experiment 1						Batch experiment 2					
	Test 1	Test 2	Test 3	Test 4	Test 5	Test 6	Test 1	Test 2	Test 3	Test 4	Test 5	Test 6
NO_2_-N (mg/L)	39.98	43.33	44.65	42.44	41.75	48.63	70.3	94.33	112.36	70	74.9	110.6
pH	6.0	6.5	7.0	7.5	8.0	8.5	7.5	7.5	7.5	7.0	7.0	7.0
*T* (°C)	24	24	24	24	24	24	19	19	19	20	20	20
FNA (mgHNO_2_-N/L)	92.3 × 10^−3^	31.6 × 10^−3^	10.3 × 10^−3^	3.10 × 10^−3^	0.96 × 10^−3^	0.35 × 10^−3^	5.87 × 10^−3^	7.88 × 10^−3^	9.38 × 10^−3^	17.81 × 10^−3^	19.06 × 10^−3^	28.14 × 10^−3^
Anoxic phase (min)	360	360	360	220	220	220	150	150	150	150	150	150
MLVSS (g/L)	2.478	2.752	2.506	2.746	2.744	2.456	2.206	2.44	2.156	2.348	2.194	1.77

**Table 2:** Various rates of denitrifying phosphorus removal by PAOs.

	FNA concentration (mgHNO_2_-N/L)	Phosphorus uptake rate (mgPO_4_-P/h·gVSS)		Nitrite reduction rate (mgNO_2_-N/h·gVSS)	The highest N_2_O to TN proportion (%)	PHA degradation rate (mmol-C/h·gVSS)	PHA consumption to TN proportion (mmol-C/mmol-N·gVSS)
		Nitrite exit conditions	Average rate of anoxic phase			Nitrite exit conditions	Average rate of anoxic phase
pH 6.0	92.3 × 10^−3^	−2.08	−2.08	1.16	18.80	−0.091	-
pH 6.5	31.6 × 10^−3^	0.53	0.53	2.14	26.81	0.067	-
pH 7.0	10.3 × 10^−3^	6.12	5.38	4.82	40.63	0.278	0.916
pH 7.5	3.10 × 10^−3^	9.66	6.13	8.15	78.42	0.589	0.546
pH 8.0	0.96 × 10^−3^	11.10	8.10	8.25	61.55	0.581	0.532
pH 8.5	0.35 × 10^−3^	15.82	8.62	11.7	44.04	0.603	0.444
